# Advanced Chemical Reduction of Reduced Graphene Oxide and Its Photocatalytic Activity in Degrading Reactive Black 5

**DOI:** 10.3390/ma8105363

**Published:** 2015-10-19

**Authors:** Christelle Pau Ping Wong, Chin Wei Lai, Kian Mun Lee, Sharifah Bee Abd Hamid

**Affiliations:** Nanotechnology & Catalysis Research Centre (NANOCAT), Level 3, Block A, IPS Building, University of Malaya (UM), Kuala Lumpur 50603, Malaysia; christellewpp.91@gmail.com (C.P.P.W.); leekianmun@um.edu.my (K.M.L.); sharifahbee@um.edu.my (S.B.A.H.)

**Keywords:** GO, rGO, Reactive Black 5, dye, photodecolorization, chemical reduction

## Abstract

Textile industries consume large volumes of water for dye processing, leading to undesirable toxic dyes in water bodies. Dyestuffs are harmful to human health and aquatic life, and such illnesses as cholera, dysentery, hepatitis A, and hinder the photosynthetic activity of aquatic plants. To overcome this environmental problem, the advanced oxidation process is a promising technique to mineralize a wide range of dyes in water systems. In this work, reduced graphene oxide (rGO) was prepared via an advanced chemical reduction route, and its photocatalytic activity was tested by photodegrading Reactive Black 5 (RB5) dye in aqueous solution. rGO was synthesized by dispersing the graphite oxide into the water to form a graphene oxide (GO) solution followed by the addition of hydrazine. Graphite oxide was prepared using a modified Hummers’ method by using potassium permanganate and concentrated sulphuric acid. The resulted rGO nanoparticles were characterized using ultraviolet-visible spectrophotometry (UV-Vis), X-ray powder diffraction (XRD), Raman, and Scanning Electron Microscopy (SEM) to further investigate their chemical properties. A characteristic peak of rGO-48 h (275 cm^−1^) was observed in the UV spectrum. Further, the appearance of a broad peak (002), centred at 2θ = 24.1°, in XRD showing that graphene oxide was reduced to rGO. Based on our results, it was found that the resulted rGO-48 h nanoparticles achieved 49% photodecolorization of RB5 under UV irradiation at pH 3 in 60 min. This was attributed to the high and efficient electron transport behaviors of rGO between aromatic regions of rGO and RB5 molecules.

## 1. Introduction

The discharge of azo dyes, which are highly stable and carcinogenic, into water bodies are harmful to human health, and cause such illness as cholera, diarrhea, amebic dysentery, and hepatitis A [1]. Dyes also affect aquatic life by hindering the photosynthesis process of aquatic plants, eutrophication, and perturbation [2,3]. Therefore, numerous techniques have been applied to treat textile wastewater, such as activated carbon adsorption (physical method), chlorination (chemical method), and aerobic biodegradation (biochemical method) [4]. However, further treatments are needed, which create secondary pollution in the environment, such as the breakdown of parent azo dyes to toxic benzidine and other aromatic compounds [5].

Advanced oxidation processes (AOPs) are widely applied to mineralize dyes into CO_2_ and H_2_O [6,7]. AOPs include ozonation, photolysis, and photocatalysis with the aid of oxidants, light, and semiconductors. Photocatalytic degradation was initiated when the photocatalysts absorb photons (UV) to generate electron-hole pairs on the catalyst surface. The positive hole in the valence band (h_VB_^+^) will reacts with water to form hydroxyl radical (·OH), followed by the oxidization of pollutants to CO_2_ and H_2_O [8].

Reactive Black 5 (RB5), also known as Remazol Black B, is an azo dye with two azo bonds (-N=N-) in its structure (Table 1). RB5 is widely used in textile industries for dye processing, and up to 50% of the dyes consumed in textile industries are azo dyes [8,9,10]. This is due to their easy preparation steps, high solubility, and stability [4]. In the past few years, several catalysts have been used to degrade RB5, such as TiO_2_ [11], BiFeO_3_ [4], ZnS [12], and ZnO [8], and the results were summarized in Table 2.

**Table 1:** Properties of Reactive Black 5 (RB5).

Properties	Anionic Azo Reactive Dye
Synonym name	Remazol Black B
Composition	≥50% of dye content
Molecular formula	C_26_H_21_N_5_Na_4_O_19_S_6_
Molecular weight	991.82 g·mol^−1^
Absorbance wavelength (λ_max_)	597 nm
Molecular Structure	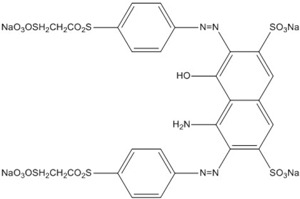

**Table 2:** The photocatalytic degradation of RB5 using several catalysts.

Authors/Year	Catalysts	Degradation Efficiency (%)	Conditions	Reference
Soltani *et al.* 2013	BiFeO_3_	99% RB5	Time: 50 min; Catalyst loading: 0.5 g·L^−1^; Irradiation: Natural Sunlight; pH 2.5	[4]
Goharshadi *et al.* 2013	ZnS	95% RB5	Time: 10 min; Catalyst loading: 0.15 g·L^−1^; Irradiation: UV light; pH 7	[12]
Laohaprapanon *et al.* 2015	ZnO	85% RB5	Time: 30 min; Catalyst loading: 1.25 g·L^−1^; Irradiation: UV light; pH 7	[13]
Lucas *et al.* 2013	TiO_2_ TiO_2_ P25	93% RB5 97% RB5	Time: 60 min; Catalyst loading: 0.1 g·L^−1^; Irradiation: UV light; pH 5.7	[11]

Reduced graphene oxide (rGO) is a 2D carbon material and is packed in a honeycomb crystal lattice. Numerous methods have been used in the synthesis of rGO, such as chemical, thermal, microwave, and microbial/bacterial [14]. Chemical exfoliation is preferable due to its large-scale production and low cost. Chemical exfoliation involves three steps, oxidation of graphite powder, dispersion of graphite oxide to graphene oxide (GO), and GO reduction to produced reduced graphene oxide (rGO) [15]. rGO has been widely applied in energy storage devices, photovoltaics devices, corrosion protection, and lubricants. This is due to its unique properties, such as high conductivity, chemical stability, high surface area (>2600 m^2^·g^−1^), high electron mobility (15,000 cm^2^·Vs^−1^), and superior thermal conductivity [16,17]. As a potential photocatalytic material, rGO has been used in the decolorization of Rhodamine B [18] and methylene blue [19] and phenol [20].

In this experiment, rGO nanoparticles were synthesized via an advanced chemical reduction method, from graphene oxide, by using hydrazine as the reducing agent. rGO was used as the photocatalysts to photodecolorize RB5 in aqueous solution under UV irradiation. To best of our knowledge, detailed investigations on catalyst loading, initial dye concentration, and initial solution pH are still lacking. This study aims to determine the optimum experimental conditions for the best photodecolorization performance.

## 2. Experimental Section

### 2.1. Chemicals and Materials

Graphite powder and hydrazine solution (35 wt% in H_2_O) were purchased from Sigma Aldrich (Steinheim, Germany). Sulfuric acid (95%–97%) was obtained from Merck (Darmstadt, Germany). Hydrochloric acid (37%) and hydrogen peroxide (30%) were gifted from R&M Chemicals (Essex, UK). Potassium permanganate was purchased from Friendemann Schmidt Chemical (Woodpark, Australia). The chemicals were used without further purifications. Reactive Black 5 (50% of dye contents) powder from Sigma-Aldrich (Steinheim, Germany) was used as the model compound in this study. Deionized water (18.2 MΩ) was used throughout the experiments.

### 2.2. Synthesis of Reduced Graphene Oxide

Graphite oxide is produced through the modified Hummers’ method by oxidizing the graphite powder [21]. In a typical synthesis, 4 g of graphite powder is mixed with 300 mL of concentrated sulfuric acid. Then, 35 g of KMnO_4_ is slowly added and stirred in an ice-bath for 4 h at 35 °C. Then, the mixture was diluted with deionized water to keep the solution below 50 °C, followed by dropwise addition of 30% H_2_O_2_. The mixture was centrifuged and washed with HCl solution to remove the metal ions and 2 L of deionized water to remove the acid. The resulting precipitates were dried in vacuum desiccators overnight.

Graphene oxide was produced by adding 1 g of graphite oxide powder into 333 mL of distilled water under mechanical stirring. Then, 0.3 mL of hydrazine was added into the mixture solution and was heated in an oil bath at 80 °C with continuous stirring for 48 h. Finally, the precipitate was washed with distilled water and oven-dried overnight.

### 2.3. Characterization

Raman spectrum was recorded using Renishaw (Gloucestershire, UK) in Via with Ar^+^ laser at 514 nm as the excitation source. The scanning range is 400–4000 cm^−1^. The X-ray diffraction pattern of rGO was recorded by a Bruker axs (Karlsruhe, Germany) D8 Advance diffractometer from 5° to 90°. The sample was scanned over Cu Kα radiation source (λ = 1.5406 Å) at 40 kV and 30 mA with a scanning rate of 0.01°·s^−1^. The surface morphology of rGO was observed by HITACHI (Tokyo, Japan) TM3030 table-top scanning electron microscope (SEM). The decolorization percentage of RB5 was determined by using an ultraviolet-visible spectrophotometer (Agilent Cary-60, Santa Clara, CA, USA) at λ_max_ = 597 nm.

### 2.4. Photocatalytic Reaction

Photocatalytic experiments were carried out by photodegrading RB5 under UV light in a custom-made photoreactor. A 96 W UV-A lamp was used as the irradiation source. In a typical experiment, 20 mg of rGO was added into a 100 mL RB5 solution (10 mg·L^−1^). The suspension was continuously stirred and was air-bubbled for 10 min in darkness to achieve adsorption-desorption equilibrium. Then, the reaction was initiated under UV light for 20 min. The samples were taken at regular time intervals and filtered using a 0.45-μm cellulose nitrate syringe filter to remove the rGO particles. The photodecolorization experiments were conducted at least three times to acquire reproducibility results. Where required, the initial pH of solution was adjusted by small amount of 0.1 M NaOH and 0.1 M HCl. The decolorization efficiency of RB5 was determined by using the equation shown below:

Decolorization efficiency (%) = (C_0_−C)/C_0_ × 100 (1)

where C_0_ is the initial concentration of RB5, C is the concentration of RB5 at time, t.

## 3. Results and Discussions

### 3.1. Characterization of Catalyst

**Figure 1:**
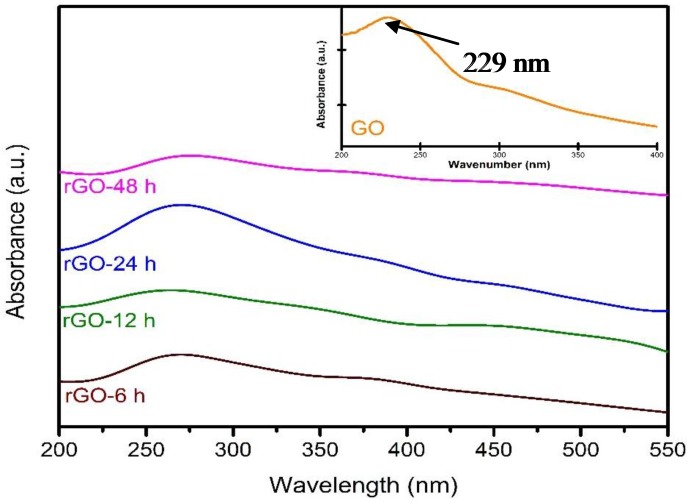
UV-vis absorption spectra of rGO with 6 h, 12 h, 24 h, and 48 h of reduction time. The insert shows the UV-vis absorption spectrum of GO.

The UV-visible absorption spectra of rGO synthesized at different reduction times are shown in Figure 1. Based on the UV-visible absorption spectra, the absorption peak of rGO at 229 nm is due to the π-π* transition of the aromatic C-C bond [22]. After the reduction treatment, obvious absorption peaks were found between 265–275 nm. It could be noticed that the absorption peak of rGO-6 h was exhibited at 269 nm. The reason for this could be attributed to the fact that the 6-h reduction time did not complete the transformation of GO into rGO. Meanwhile, the peak at 269 nm was red shifted to 275 nm after 48 h of reduction time [23]. It is well known that the red shift in the UV-visible absorption spectra is mainly due to the restoration of the sp^2^ hybridization carbon atoms and increase of electron concentration [24].

**Figure 2:**
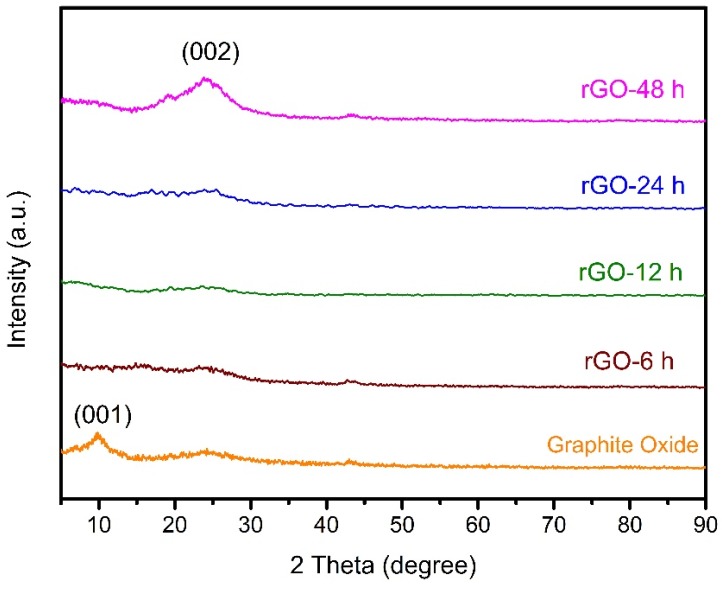
XRD pattern of graphite oxide, rGO-6 h, 12 h, 24 h, and 48 h.

The XRD patterns of graphite oxide in Figure 2, showed that a strong diffraction peak at 9.95° corresponds to (001) plane [25] and a weak diffraction peak is observed at 24.1°, owing to the deoxygenation of weak oxygen functional groups and restacking of graphene layers during the drying process of graphite oxide [26]. After advanced chemical reduction, the peak at 9.95° disappeared and the intensity of rGO peak (002) increased with an increase in reduction time, from 6 h up to 48 h. This shows that the graphite oxide had been effectively reduced to rGO. rGO-48 h with the highest intensity positioned at 2θ = 24.1° was detected [27], indicating the overlapping of rGO layer sheets. Wang *et al.* [28] claimed that the overlapping or restacking of rGO sheets enhanced the electronic conductivity of rGO. The broad peak of rGO-48 h reflects its low crystallinity index [29].

**Figure 3:**
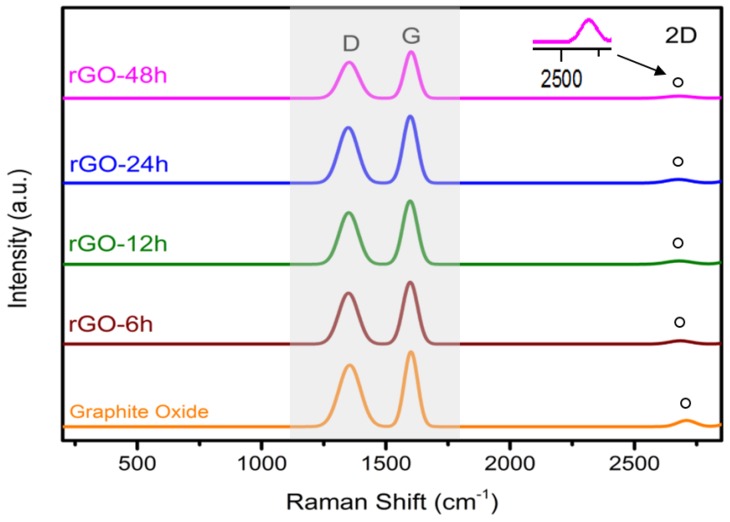
Raman spectra of graphite oxide, rGO-6 h, 12 h, 24 h, and 48 h.

In Figure 3, the Raman spectra of rGO contain two prominent peaks, namely D band and G band, in the range of 1349–1352 cm^−1^ and 1601–1604 cm^−1^. For graphite oxide, the D and G bands appear at 1355 cm^−1^ and 1605 cm^−1^, respectively. These characteristic peaks represent the defects in carbon atoms and the scattering of E_2g_ phonon of sp^2^ carbon atoms [24,30]. The intensity ratio of D and G bands (I_D_/I_G_) is used to determine defects of graphene materials. The I_D_/I_G_ of graphite oxide, rGO-6 h and rGO-48 h are 0.85, 0.85, and 0.88, respectively. rGO-48 h has the highest I_D_/I_G_ ratio compared to that of graphite oxide and rGO-6 h, due to a defect after the oxygen groups are removed and introduction of large amounts of sp^2^ carbon networks with small average sizes [31,32]. The 2D band is the key features to verify the number layers of graphene sheets in Raman Spectroscopy. The 2D band of rGO-6 h and 48 h was located at 2686 cm^−1^ and 2680 cm^−1^, while the 2D band of graphite oxide was positioned at 2706 cm^−1^. The 2D band of rGO-48 h peak was shifted to a lower wave number than rGO-6 h and graphite oxide, indicating that the rGO-48 h contains fewer graphene layers than rGO-6 h and graphite oxide [33].

**Figure 4:**
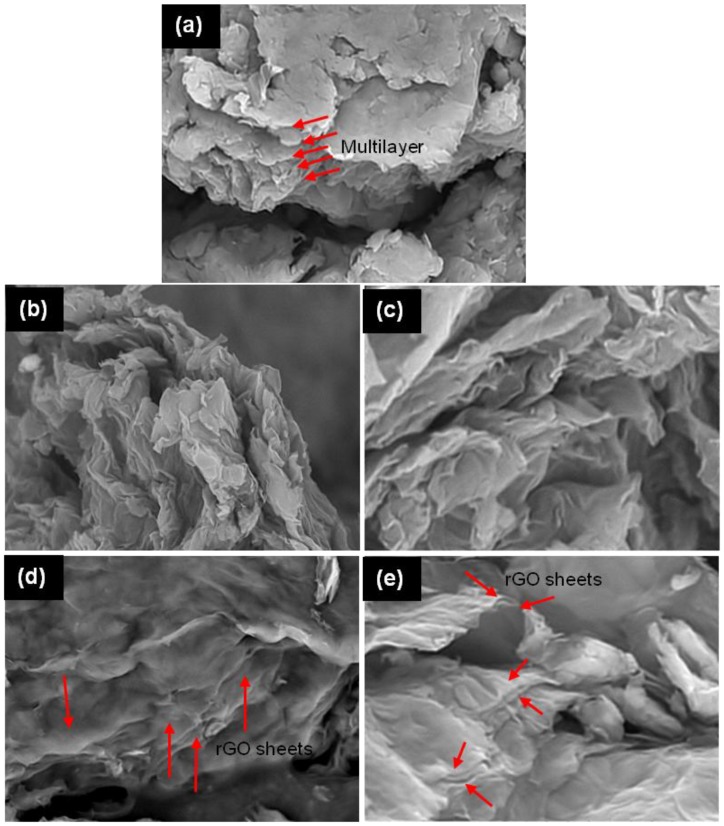
SEM image of (**a**) graphite oxide and rGO at different reduction time; (**b**) 6 h; (**c**) 12 h; (**d**) 24 h; and (**e**) 48 h.

Figure 4 presents the SEM images of graphite oxide and rGO at different reduction times. Figure 4a shows that graphite oxide has a multilayer structure and the oxygen functional groups have prevented it from stacking. After 6 h of reduction, the rGO was still in an agglomeration form with a rough and cracked surface, as shown in Figure 4b. This reason is mainly attributed to the insufficient reaction time to remove the oxygen functional groups that attached at the edge and basal plane of the graphene layer. rGO-24 h and -48 h has a layer structured with lots of wrinkles, which overlapped with each other instead of aggregating [34].

### 3.2. Reduction Time

**Figure 5:**
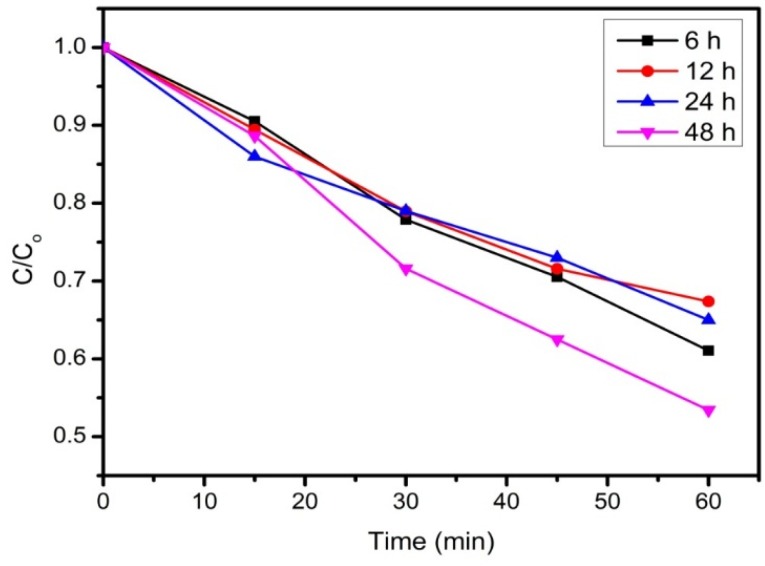
Photodecolorization of RB5 by rGO-6 h, rGO-12 h, rGO-24 h and rGO-48 h under UV irradiation. Conditions: (RB5) = 10 mg·L^−1^, rGO loading = 0.2 g·L^−1^, pH = 4.

The effect of reduction time of rGO on the photodecolorization of RB5 was studied. As shown in Figure 5, rGO-48 h exhibited the highest photodecolorization efficiency due to its higher level of reduction. Mamba *et al.* [35] claimed that the degree of reduction will influence the role of rGO as an electron acceptor and charge shuttling activities. As is well known, graphite oxide is an insulator and rGO is a conducting material owing to the restoration of sp^2^ hybridization during the reduction process. rGO has superior electronic conductivity due to its sp^2^ hybridization, which restrained the recombination of electron-hole pairs during photodecolorization [17]. Hence, rGO-48 h will be used in the following section.

### 3.3. Effect of rGO Loading

**Figure 6:**
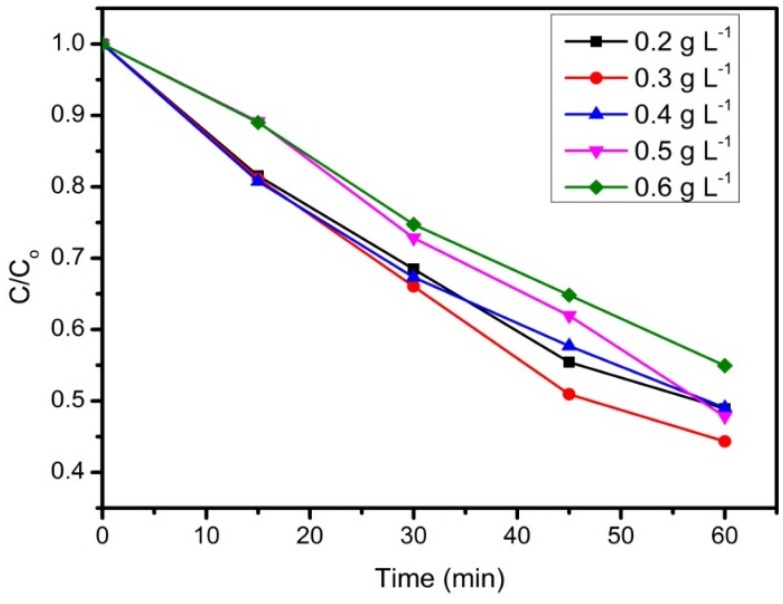
The effect of rGO loading on photodecolorization of RB5. Conditions: (RB5) = 10 mg·L^−1^, pH = 4.

Figure 6 shows the effect of different rGO loadings on the photodecolorization of RB5. It can be observed that the maximum photodecolorization efficiency of 56% was achieved with 30 mg of rGO loaded after 60 min irradiation. It has been reported that an increase in the catalyst dosage enhanced the decolorization efficiency, as more active sites are available on the catalyst surface to absorb dye molecules [36,37]. Nonetheless, the concentration of RB5 is hardly reduced at higher catalyst loadings. This is possibly due to the agglomeration of particles, which reduced the surface area of catalysts for dye adsorption. Moreover, the penetration of UV light into the suspension was hindered as the solutions become turbid at higher loadings [38,39].

### 3.4. Effect of RB5 Concentration and Its Kinetics

**Figure 7:**
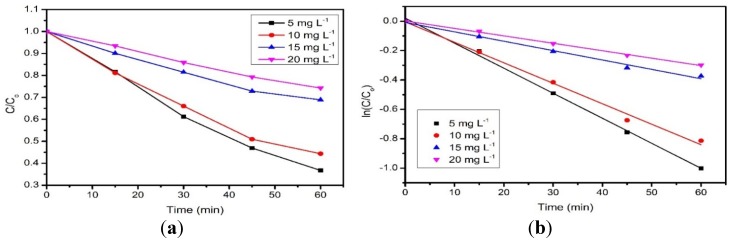
(**a**) Photodecolorization of RB5 with varying RB5 concentration; (**b**) Plot of ln(C/C_0_) as a function of rGO at different RB5 concentration. Conditions: rGO loading = 0.3 g·L^−1^, pH = 4.

Photodecolorization of RB5 was examined by using different initial concentrations of RB5, ranging from 5 to 20 mg·L^−1^ with 30 mg of rGO. Figure 7a shows that the photodecolorization efficiency of RB5 is inversely proportional to the concentration of RB5. As the initial concentration of RB5 increased, the photodecolorization efficiency of RB5 dramatically declined from 63% to 26% after 60 min of irradiation. The excessive amounts of dye molecules attached on the rGO surface may block the photon from reaching the catalyst surface, which resulted in lower decolorization efficiency [12,13].

The RB5 decolorization was fitted to pseudo-first order kinetics by referring to the Langmuir-Hinshelwood kinetic model (Equation (2)) [17,40]:

(2) $$\ln\left(\frac{\text{C}}{{\text{C}}_{\text{o}}}\right)=-\text{kt}$$

where C is the concentration of RB5 at time, t, ${\text{C}}_{\text{o}}$ is the initial concentration of RB5, and k is the pseudo-first order rate constant. The k value of respective concentrations was determined from the slope of the linear plot (Figure 7b) and was listed in Table 3. The value of k progressively reduced with increasing RB5 concentrations [41]. The correlation coefficient (R^2^) values are close to 1, which obeys the pseudo-first order kinetic model.

**Table 3:** Degradation efficiency and pseudo-first order rate constant for photocatalytic degradation of RB5 by rGO under UV irradiation.

Concentration of RB5 (mg/L)	Degradation efficiency (%)	R^2^	Value of k (min^−1^)
5	63	0.99684	0.01704
10	56	0.99188	0.01395
15	31	0.98723	0.00639
20	26	0.99768	0.00507

### 3.5. Effect of pH

**Figure 8:**
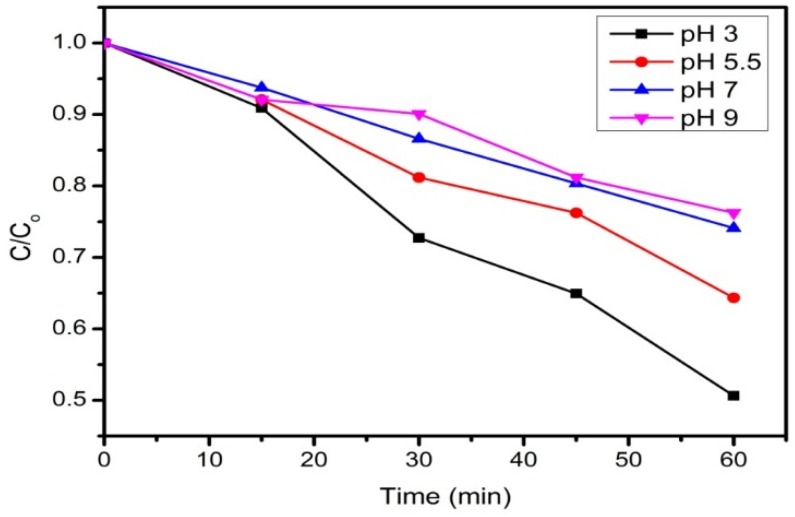
The photodecolorization of RB5 in different initial pH solutions. Conditions: (RB5) = 10 mg·L^−1^, rGO loading = 0.3 g·L^−1^.

The effect of pH on the photodecolorization of RB5 was studied at different pH values, *i.e.*, 3, 5.5, 7, and 9. Figure 8 shows that the photodecolorization of RB5 in acidic pH is better than in basic and neutral pH. A possible explanation is the pKa value of RB5 is low, due to the sulfonate groups that are attached to the dye molecule [42]. Hence, at a lower pH, there is an electrostatic attraction between the negatively charge of RB5 and the positively charge of rGO nanoparticles [43], which resulted in a higher photodecolorization percentage.

## 4. Conclusions

In summary, rGO nanoparticles were successfully synthesized via an advanced and simple chemical reduction method. The potential of rGO nanoparticles as a photocatalyst was evaluated by photodecolorization of RB5 under UV-A irradiation. Based on our studies, rGO-48 h showed higher photocatalytic activity than rGO that was prepared in 6 h, 12 h, and 24 h, owing to the high overlapping of rGO layers structure with enhanced electronic conductivity. The presence of a sp^2^ carbon structure could improve the ability of electron transport between rGO and RB5 dye, which further minimizes the recombination losses of charge carriers. Interestingly, it was found that an optimum experimental condition for the effective removal of RB5 (49%) was 30 mg rGO loading, 10 mg·L^−1^ of RB5 and at a solution pH of 3.
